# A unique environmental augmented household-level livelihood panel dataset from Nepal

**DOI:** 10.1016/j.dib.2022.108168

**Published:** 2022-04-14

**Authors:** Solomon Zena Walelign, Carsten Smith-Hall, Santosh Rayamajhi, Bir B.K. Chhetri

**Affiliations:** a Department of Geography, Faculty of Social and Educational Sciences, Norwegian University of Science and Technology, Bygg 7, Dragvoll, Norway; b School of Economics, College of Business and Economics, University of Gondar, Gondar, Ethiopia; c Department of Food and Resource Economics, Faculty of Science, University of Copenhagen, Rolighedsvej 23, Frederiksberg C, 1958, Denmark; d Institute of Forestry, Tribhuvan University, Kirtipur, Nepal

**Keywords:** Assets, Dynamics, Environment, Income, Panel data, Livelihoods, Poverty, South Asia

## Abstract

This paper presents primary household-level panel data for the investigation of rural livelihoods dynamics in Nepal. The data is environmental augmented through the inclusion of information on environmental resource use allowing estimation of household-level environmental income. The main variables included are: household demographics (individual's age, gender, educational status, marital status), assets (livestock, implements, land, jewellery, saving, debt), income (from the environment, crop production, livestock rearing, business ownership, wage employment, remittances, and other sources), and household shock experiences (e.g., crop failure or livestock loss). Spanning the three main physiographic regions in Nepal, data was collected in the districts of Chitwan (lowland), Kaski (mid-hills), and Mustang (mountains) in 2006 (*n* = 507), 2009 (*n* = 446), and 2012 (*n* = 428), with households randomly sampled, using trained and monitored enumerators. The structured household survey is freely available in Larsen et al. (2014) that also provides complete data collection process details. In each study year, household income data were collected quarterly (using recall periods of 1 or 3 months, depending on the product), while asset data was collected twice (at the beginning and end of each year). Farm-gate prices were used to value products whenever possible; subsistence products were valued using substitute product prices or the opportunity cost of time (i.e., local wage labour rate). Basic distributional statistics indicated that estimated values have acceptable properties allowing their use as prices. The dataset can be reused for analyses across a range of topics (e.g. focused on forests or livestock), data types (e.g. income or asset), and temporal scales (static or selected years).

**Specifications Table**

**Table:** 

Subject	Economics
Specific subject area	The dynamics of household-level absolute and relative environmental income, livelihoods, and rural poverty
Type of data	Tables Figures
How data were acquired	The structured household survey, described and available at https://static-curis.ku.dk/portal/files/125231441/IFRO_Documentation_2014_4.pdf
Data format	Raw Filtered
Parameters for data collection	The study locations span the main physiographic regions of Nepal, i.e., the lowlands, mid-hills, and mountains. Selection criteria were: (i) the altitudinal and vegetation variations in Nepal, (ii) households' environmental reliance, (iii) communities' attitudes toward long-term research, and (iv) village accessibility and researcher safety (due to the civil war in Nepal during site selection in 2005).
Description of data collection	Spanning the three main physiographic regions in Nepal, data was collected in the districts of Chitwan (lowland), Kaski (mid-hills), and Mustang (mountains) in 2006 (n=507), 2009 (n=446), and 2012 (n=428), with households randomly sampled, using trained and monitored enumerators. In each study year, household income data were collected quarterly (using recall periods of 1 or 3 months, depending on the product), while asset data was collected twice (at the beginning and end of each year). Farm-gate prices were used to value products whenever possible; subsistence products were valued using substitute product prices or the opportunity cost of time (i.e., local wage labour rate).
Data source location	Institution: Institute of Forestry City/Town/Region: Kirtipur, Kathmandu Country: Nepal Latitude and longitude for collected data: a. In Chitwan District, Chainpur: 27°37′57″N and 84°33′46″E. b. In Kaski District, Hemja: 28°14′48″N - 28°18′5″N and 83°52′46″E – 83°58′18″E. c. In Mustang District, Lete and Kunjo: 28°34′-28°41′ N and 83°33′-83°44′ E.
Data accessibility	The dataset and accompanying files are freely available and hosted on Mendeley Repository name: Mendeley Data identification number: doi: 10.17632/k5b2zmr4kp.2 Direct URL to data: https://data.mendeley.com/datasets/k5b2zmr4kp/2
Related research article	Walelign, S.Z, Pouliot, M., Larsen, H.O. and Smith-Hall, C. 2017. Combining household income and asset data to identify livelihood strategies and their dynamics. Journal of Development Studies 53(6):769-787.

**Value of the Data**

• There are no other published household-level panel datasets that include environmental resource data, allowing estimation of environmental income and thus providing more accurate total household income estimates.
• The dataset is of high interest to researchers and students delving into dynamic livelihood and poverty analyses, particularly to understand the role and potential of environmental products.
• The data can be used to test a string of assumptions regarding the economic importance of environmental products, including their changes over time.
• In-depth dataset descriptions (including data collection and quality checks) allow for establishing comparative studies across other sites.

## 1 Data Description

**Fig. 1:**
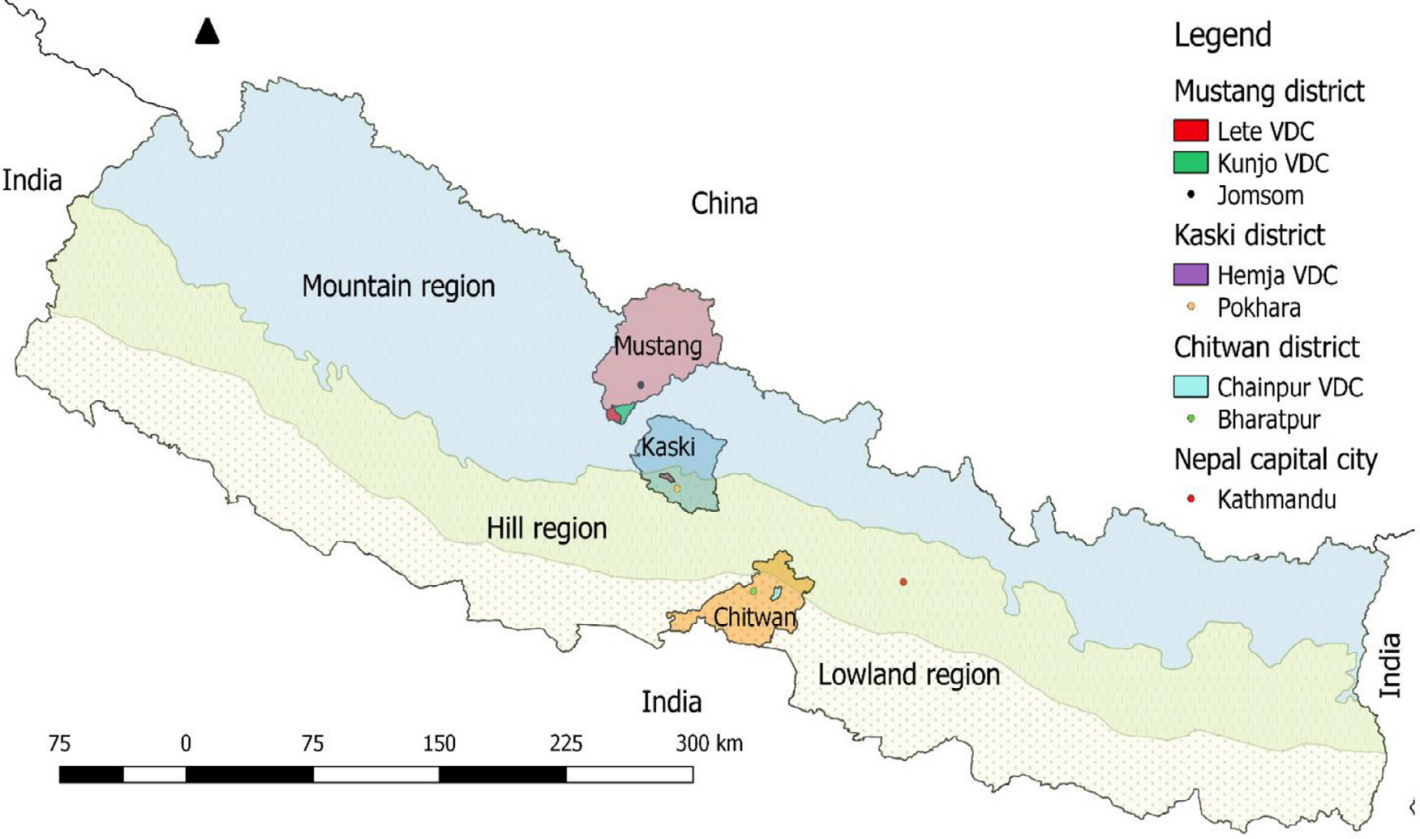
Location of study sites in Nepal [2].

The dataset is environmentally augmented, containing information on each household's environmental resource use which allows estimation of environmental income as part of total household income The location of the study sites (districts and VDCs) is shown in Fig. 1, along with the three major physiographic zones of Nepal.

Table 1 presents the household-level data for mean income from each income source (and yearly totals) by study year (2006, 2009, and 2012) and district (Chitwan, Kaski, and Mustang). We used the eight income categories (environment, crop, livestock, remittances, support, business, wage, and other) described by Charlery and Walelign [3]. The first three income sources encompass both cash and subsistence income (the latter is the value of environmental resources consumed at home, i.e. not sold). The income values are adjusted for adult equivalent units [4]. All values are expressed in nominal Nepalese rupees.

**Table 1:** Household-level mean income by year, district, and source (all figures in nominal Nepalese rupees, NR, per adult equivalent unit; standard deviation in brackets).

		Environmental Income	Crop Income	Livestock Income	Remittances	Support Income	Business Income	Wage Income	Other Income	Total Income
2006	Chitwan	1603 (1762)	3379 (5231)	4692 (6838)	7068 (21941)	2273 (5008)	3286 (19836)	593 (1063)	1150 (4643)	24044 (35654)
	Kaski	5705 (4465)	8012 (7628)	5500 (5250)	8670 (25742)	5770 (8285)	-3466 (44571)	180 (418)	2032 (7923)	32403 (53492)
	Mustang	17695 (17248)	4875 (6477)	11882 (32829)	4538 (14647)	839 (2512)	29698 (68299)	1905 (4437)	6302 (13505)	77735 (85072)
	Overall	8429 (12914)	4969 (6527)	7511 (20746)	6500 (20616)	2533 (5594)	11457 (50091)	982 (2866)	3239 (9749)	45621 (66419)
2009	Chitwan	8481 (67292)	7223 (7212)	7584 (16915)	16225 (34087)	5139 (9711)	-1842 (55655)	1330 (3092)	1927 (18093)	46067 (109110)
	Kaski	6934 (8746)	14416 (12833)	8249 (13108)	13108 (38017)	18112 (26132)	11899 (37621)	366 (741)	15287 (113190)	88370 (136450)
	Mustang	9943 (15743)	12316 (14989)	13998 (21846)	12701 (25986)	6550 (11266)	20723 (377126)	1003 (2299)	5181 (15239)	82414 (377945)
	Overall	8638 (44809)	10650 (12111)	9979 (18282)	14279 (32470)	8600 (16313)	9193 (226410)	995 (2474)	6121 (56154)	68455 (243626)
2012	Chitwan	5486 (5247)	532 (5933)	5945 (59030)	16565 (51148)	2451 (7198)	4866 (108722)	2158 (5699)	9041 (28871)	47045 (140269)
	Kaski	11283 (7651)	8685 (53180)	15852 (21818)	18826 (56381)	10797 (19059)	79659 (317707)	1038 (3585)	34373 (163959)	180513 (385198)
	Mustang	20785 (29309)	4442 (8545)	9120 (11999)	10736 (29671)	5788 (15375)	30065 (71112)	2342 (5396)	18812 (48184)	102091 (104829)
	Overall	12084 (19087)	3749 (26347)	9312 (40657)	15077 (46344)	5515 (14007)	30707 (174859)	1965 (5193)	18219 (85832)	96627 (220433)

Table 2 presents the household-level mean data for asset values by year, district, and type (including yearly totals). We used the nine asset types (livestock, implements, land, bank saving, jewellery, male adult household members, female adult household members, head education, and maximum household education) described by Charlery and Walelign [3]. Values related to money (total livestock, total implements, bank savings, and jewellery) are in nominal Nepalese Rupees. Values for total livestock, implements, land, bank savings, and jewellery are adjusted for adult equivalent units.

**Table 2:** Household-level mean value by year, district, and type (in nominal Nepalese Rupees, NR, per adult equivalent unit where relevant; standard deviations in brackets) of assets by year and district.

		Total Livestock	Total Implements	Total Land (in m^2^)	Bank Savings	Jewellery	No. of Male Adults	No. of Female Adults	Head Education (yrs)	Maximum Household Education (yrs)
2006	Chitwan	19139 (17910)	4682 (10883)	1918 (5999)	834 (2523)	0 (0)	1.79 (1.14)	1.80 (1.01)	3.14 (4.13)	8.53 (3.82)
	Kaski	25415 (20471)	17379 (31995)	1289 (1489)	11445 (28902)	0 (0)	1.62 (0.96)	1.82 (1.05)	6.00 (4.99)	10.57 (3.14)
	Mustang	84567 (248190)	10199 (19214)	2836 (2702)	31703 (77090)	37043 (92242)	1.62 (1.09)	1.49 (0.94)	3.00 (3.87)	7.30 (3.54)
	Overall	44554 (153885)	9561 (20868)	2113 (4263)	14545 (50490)	13590 (58568)	1.69 (1.09)	1.69 (1.00)	3.73 (4.41)	8.54 (3.77)
2009	Chitwan	45057 (40109)	10259 (23702)	944 (782)	1989 (6211)	4758 (7528)	1.78 (1.12)	1.94 (0.98)	2.91 (4.08)	9.78 (3.62)
	Kaski	34503 (35588)	31016 (45495)	1472 (2026)	12896 (31638)	20962 (16688)	1.72 (1.03)	1.75 (0.97)	6.10 (5.21)	11.13 (3.91)
	Mustang	66536 (204599)	15684 (25787)	2517 (5575)	24199 (57929)	38005 (77311)	1.67 (1.03)	1.63 (0.94)	3.01 (3.78)	8.06 (3.90)
	Overall	50156 (125321)	16904 (31659)	1615 (3534)	12252 (38819)	20093 (48794)	1.73 (1.07)	1.79 (0.97)	3.68 (4.46)	9.49 (3.96)
2012	Chitwan	37987 (31602)	22156 (38194)	1032 (1133)	12019 (31839)	21096 (23113)	1.71 (1.12)	1.96 (1.06)	2.92 (4.34)	9.95 (4.39)
	Kaski	34636 (39307)	48959 (70583)	1375 (2254)	25411 (66932)	51606 (48464)	1.81 (1.12)	1.89 (1.02)	6.91 (5.07)	11.91 (4.03)
	Mustang	34115 (39336)	21467 (27566)	1921 (1893)	48051 (104495)	54132 (112328)	1.73 (1.27)	1.58 (1.00)	2.90 (4.08)	8.22 (3.87)
	Overall	35885 (36191)	28070 (46314)	1417 (1755)	27497 (73771)	39471 (73065)	1.74 (1.17)	1.81 (1.04)	3.83 (4.73)	9.80 (4.35)

The complete dataset (named “ComForM_Panel_data”) is deposited on Mendeley Data in two versions: Stata (.dta) and csv data formats. The dataset contains information on 91 variables. The main variables are: household demographics (individual’s age, gender, educational status, marital status), assets (livestock, implements, land, jewellery, saving, debt), income (from the environment, crop production, livestock rearing, business ownership, wage employment, remittances, and other sources), and household shock experiences (e.g., crop failure or livestock loss). The variables in the data are labelled. We also deposited an Excel file named "Data dictionary" that provides the full description of the variables. The associated complete questionnaire is available in [1].

In the dataset, note that (i) household demographic variables (e.g., number of households members in different age groups) has been collapsed to the household level, and (ii) variables collected quarterly (income and livestock assets) were aggregated to annual values. The raw dataset is available upon request from the corresponding author.

## 2 Experimental Design, Materials and Methods

The study site selection criteria were: (i) the altitudinal and vegetation variations in Nepal, (ii) households’ environmental reliance, (iii) communities’ attitudes toward long-term research, and (iv) village accessibility and researcher safety (due to the civil war in Nepal during site selection in 2005). Following extensive site visits, study sites were selected in Lete and Kunjo Village Development Committees (VDCs, the lowest administrative unit) in Mustang District in the Himalayas, Hemja VDC in Kaski District in the mid-hills, and Chainpur VDC in Chitwan District in the lowlands [5]. For detailed site descriptions, see [1].

Data collection was organised and conducted as part of the Community Based Forest and Tree Management in the Himalaya (ComForM) project, which collaborated with the Poverty Environment Network (PEN) to develop the structured household survey. Based on the PEN guidelines and prototype questionnaire [6,7], a site and project-specific version were developed and applied in the study sites [1], resulting in the three-wave panel dataset collected in 2006, 2009, and 2012. Household income data were collected quarterly (four visits per year) to facilitate better recall and capture seasonal variation in income sources. Household demographics were collected at the beginning of each survey year. Asset data were collected at the beginning and end of each year, while household shock experiences were collected at the end of each year. The 2006 data was collected from 507 randomly selected households across the three districts in the four sites: *n* = 114 in Hemja, representing about 6.0% of the population in 2001; *n* = 207 in Chitwan (7.4%); *n* = 88 in Kunjo (57.9%) and *n* = 98 in Lete (44.0%). The sampling frame in Lete and Kunjo was the latest census list of households provided by the Village Development Committees while the sampling frame in Hemja and Chainpur was the respective forest user groups (FUGs) member lists. All sampling frames were checked and updated using key informants.

Of the original 507 households, 446 were resurveyed in 2009 and, of these, 428 in 2012. The resultant attrition rate was 12% between 2006 and 2012, 4% between 2009 and 2012, and 16% over the six years. Analysis of the effect of attrition, based on dynamic and static attrition tests using appropriate models (e.g. probit), indicated no bias on the estimates [8].

Income was defined as the value-added of labour and capital, and environmental income was income generated by extracting products from non-cultivated sources, e.g. forests, grasslands, bushlands, wetlands, fallows, and wild plants and animals harvested from croplands [6]. This is net income – the total annual value of cash and subsistence income less the cost of all inputs except family labour. The latter is difficult to estimate, and it is standard procedure to omit from registration. All goods produced or collected by the household and used for home consumption (subsistence) were valued and counted as part of household income. Absent local market prices, we used alternative valuation methods following [9]. All assets shared by household members in production and consumption (e.g. value of implements, land holding) and all income values were divided by adult equivalent units (aeu) in the household to allow inter-household comparisons.

For a site-level example of methods applied, data quality checks for households' own-reported and analytically imputed values, and the basic distributional statistics indicating that estimated values have acceptable properties allowing their use as prices, see [10].

## Ethics Statement

The research was implemented by Tribhuvan University's Institute of Forestry and the University of Copenhagen's Department of Food and Resource Economics, fulfilling all ethical requirements at both institutions. The project did not require the formal approval of the institutions’ ethical bodies. The research adhered to the European Code of Conduct for Research Integrity, the University of Copenhagen Rules on Good, Scientific Practice, and the ethical standards of the International Society for Ethnobiology. All necessary research permits were obtained. With every individual participant, we clarified the purpose and use of the research. All interviewees gave their prior informed consent to participation and were informed that they could withdraw at any point. No individual participants can be identified. Research findings were shared with all the local communities where data was collected.

## CRediT authorship contribution statement

**Solomon Zena Walelign:** Data curation, Project administration, Writing – original draft. **Carsten Smith-Hall:** Conceptualization, Methodology, Data curation, Writing – original draft, Supervision. **Santosh Rayamajhi:** Data curation, Writing – review & editing. **Bir B.K. Chhetri:** Writing – review & editing.

## Declaration of Competing Interest

The authors declare that they have no known competing financial interests or personal relationships which have or could be perceived to have influenced the work reported in this article.

## Data Availability

A unique environmental augmented household-level livelihood dataset from Nepal (Original data) (Mendeley Data).
